# Association between Pri-miR-34b/c rs4938723 polymorphism and bladder cancer risk

**DOI:** 10.7555/JBR.31.20170044

**Published:** 2017-10-10

**Authors:** Mohammad Hashemi, Vahed Hasanpour, Hiva Danesh, Fatemeh Bizhani, Behzad Narouie

**Affiliations:** 1. Cellular and Molecular Research Center; 2. Department of Clinical Biochemistry, School of Medicine; 3. Student Research Committee, Zahedan University of Medical Sciences, Zahedan, Sistan and Baluchistan 98167-43181, Iran; 4. Urology and Nephrology Research Center, Department of Urology, Shahid Labbafinejad Medical Center, Shahid Beheshti University of Medical Sciences, Tehran, Tehran 198396-3113, Iran.

**Keywords:** Pri-miR-34 b/c, bladder cancer, polymorphism, microRNA

## Abstract

Several studies examined the impact of miR-34b/c rs4938723 polymorphism and cancer risk, but the findings are inconsistent. However, no study has been conducted to inspect the impact of miR-34b/c polymorphism on bladder cancer. This study aimed to assess possible association between rs4938723 polymorphism and bladder cancer risk. This case-control study was done on 136 pathologically proven bladder cancer patients and 144 controls. Genotyping of Pri-miR-34b/c rs4938723 polymorphism was achieved by using the polymerase chain reaction restriction fragment length polymorphism (PCR-RFLP) method. Our findings did not show any statistically significant differences in genotype and allele frequencies between bladder cancer and controls. Larger sample sizes with diverse ethnicities are required to validate our findings.

## Introduction

Bladder cancer is the ninth most common malignancy in the world, and the fourth most common cancer in the United States^[1]^. Men are more than four times more likely to get bladder cancer than women. Bladder cancer has a multifactorial etiology. It has been proposed that the development of bladder cancer is a result of environmental factors such as smoking, occupational exposure to carcinogens, obesity, physical inactivity^[2–4]^, genetic variants and the interaction of genes with the external factors^[5–8]^.

MicroRNAs (miRNAs) are a class of small single-stranded noncoding RNA molecules that play key roles in a variety of cellular processes by targeting mRNAs for cleavage or translational repression^[9–10]^. The data provides strong evidence that dysregulation of miRNAs expression affects the tumorigenesis by acting as oncogenes or tumor suppressors^[11–15]^. Single-nucleotide polymorphisms (SNPs) in miRNAs can affect cancer susceptibility by disturbing miRNAs biosynthesis and expression, altering mature miRNAs, or by combining with target genes^[16–19]^.

The miR-34 family members comprises miR-34a, miR-34b, and miR-34c that are encoded by two different primary miRNAs. The miR-34a is encoded by its own transcript, while the miR-34b and miR-34c are encoded by a shared primary transcript (pri-miR-34b/c)^[20]^. A potentially functional rs4938723 variant (T to C substitution), located in the promoter region of pri-miR-34b/c, may affect miR-34b/c expression via genetic and epigenetic mechanisms and in turn influence the individual susceptibility to cancer^[21–23]^. Though the association between miR-34b/c rs4938723 polymorphism and the risk of developing several cancers were reported in various case-control studies^[20,23–34]^, but to the best of our knowledge, there is no report concerning the impact of miR-34b/c rs4938723 variant on the risk of bladder cancer. Accordingly, this case-control study was aimed to evaluate the possible association between pri-miR-34b/c rs4938723 polymorphism and susceptibility to bladder cancer in a sample from the Iranian population.

## Subjects and methods

### Patients

A total of 136 patients with histopathologically confirmed papillary urothelial cancers of the bladder and 144 healthy controls were enrolled in this case-control study. The study design and recruitment procedures were described previously^[35]^. All participants were from the Department of Urology, Shahid Labbafinejad Medical Center, Shahid Beheshti University of Medical Sciences, Tehran, Iran. The local ethics committee of Zahedan University of Medical sciences approved the project and informed us that written consent was obtained from all of the study participants. The genomic DNA was extracted from peripheral blood cells using the salting-out method^[36]^.

### Genotyping

**Fig.1:**
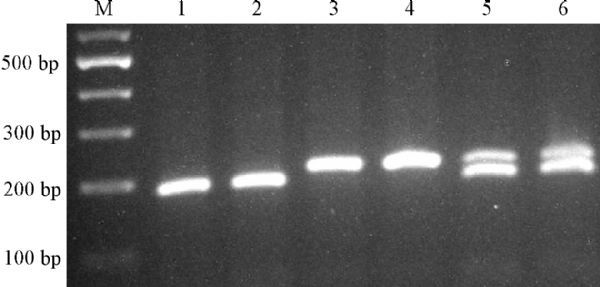
**Genotypes of pri-miR-34b/c rs4938723.** M: DNA marker; lanes 1, 2: CC genotype; lanes 3, 4: TT genotype. lane 5, 6: TC genotype.

Genotyping of the Pri-miR-34b/c rs4938723 polymorphism was done by a polymerase chain reaction-restriction fragment length polymorphism (PCR-RFLP) technique as explained previously^[37]^. Briefly, the forward and reverse primers to amplify the Pri-miR-34b/c gene sequences containing the rs4938723 polymorphisms were 5′-CCTCTGGGAACCTTCTTTGACCTGT-3′ and 5′-CCTGGGCCTTCTAGTCAA-ATAGTGA-3′, respectively. PCR amplification was done using Prime Taq Premix (Genet Bio, Korea) by the following amplification procedure: denaturation at 95 °C for 5 minutes, followed by 30 cycles of 95 °C for 30 seconds, 57 °C for 30 seconds, 72 °C for 30 seconds, with a final extension of 10 minutes at 72 °C. Ten microliters of the PCR products of 212 bp fragments were digested by NmuCI restriction enzyme (Fermentas) and separated by 2.5% agarose gel electrophoresis. The T allele was undigested (212 bp fragment), but the C allele digested and produced two fragments of 186 and 26 bp **( *Fig. 1*).**

### Statistical analysis

Statistical analysis was carried out using statistical package SPSS 22 software. The categorical and continuous data were analyzed using χ^2^ and *t*-test, respectively. Odds ratio (OR) and 95% confidence interval (CI) was computed by unconditional logistic regression analysis to determine the association between the variant and the bladder cancer. The statistical level of significance was set as the *P*<0.05 level.

## Results

The miR-34b/c rs4938723 *t*>C polymorphism was successfully genotyped for 136 bladder cancer patients (117 males, 19 females) with an average age of (63.8±12.3) years and 144 controls (134 males, 10 females) with mean age of (64.3±10.2) years. No significant difference was found between the groups regarding age ( *P* = 0.701) and sex ( *P*= 0.076). The genotype and allele frequencies of pri-miR-34b/c rs4938723 polymorphism in bladder cancer patients and controls are shown in ***Table 1***. The results indicated that pri-miR-34b/c rs4938723 *t*>C polymorphism was not associated with the risk of bladder cancer in codominant (OR= 1.08, 95% CI= 0.66–1.78, *P*= 0.800; OR= 2.13. 95%CI= 0.91–5.01, *P*= 0.094, TC *vs*. TT), dominant (OR= 1.22, 95%CI= 0.76–1.95, *P*= 0.468, TC+ CC *vs.* TT), recessive (OR= 2.04, 95%CI= 0.91–4.60, *P*= 0.110, CC *vs*. TT+ TC), overdominant (OR= 0.94, 95%CI= 0.59–1.50, *P*= 0.812, TC *vs.* TT+ CC) and allelic (OR= 1.28, 95%CI= 0.90–1.82, *P*= 0.181, C *vs*. T) inheritance model tested. We also calculated adjusted OR and 95%CI for sex and age **( *Table 1*)**. The findings revealed that the variant was not associated with bladder cancer risk.

**Tab.1:** Association between pri-miR-34b/c rs4938723 *t*>C polymorphism and risk of bladder cancer

rs4938723	Case *n* (%)	Control *n* (%)	OR (95%CI)	P	*OR (95%CI)	P
Codominant						
TT	54 (39.7)	64 (44.4)	1.00	-	1.00	-
TC	64 (47.1)	70 (48.6)	1.08 (0.66–1.78)	0.800	1.06 (0.64–1.77)	0.813
CC	18 (13.2)	10 (6.9)	2.13 (0.91–5.01)	0.094	2.11 (0.89–4.99)	0.089
Dominant						
TT	54 (39.7)	64 (44.4)	1.00	-	1.00	-
TC+ CC	82 (60.3)	80 (55.5)	1.22 (0.76–1.95)	0.468	1.19 (0.74–1.93)	0.474
Recessive						
TT+ TC	118 (86.8)	134 (93.1)	1.00	-	1.00	-
CC	18 (13.2)	10 (6.9)	2.04 (0.91–4.60)	0.110	2.04 (0.90–4.63)	0.086
Overdominant						
TT+ CC	72 (52.9)	74 (51.4)	1.00	-	1.00	-
TC	64 (47.1)	70 (48.6)	0.94 (0.59–1.50)	0.812	1.09 (0.67–1.75)	0.732
Allele						
T	172 (41.9)	198 (68.8)	1.00	-	-	-
C	100 (58.1)	90 (31.2)	1.28 (0.90–1.82)	0.181	-	-

*adjusted for sex and age

The association between pri-miR-34b/c rs4938723 polymorphism and clinicopathological characteristics of bladder cancer patients are shown in ***Table 2***. The findings propose a significant association between age and rs4938723 variant so that the TT genotype frequencies was significantly higher in patients with ages>60 years (48.2%) than that of patients with ages≤60 years (26.4%).

**Tab.2:** Association of rs4938723 polymorphism of Pri-miR-34b/c gene with clinicopathological characteristics of bladder cancer patients.(*n*)

Factors	rs4938723 *t*>C			*P*-value
	TT	TC	CC	
Age at diagnosis (years)				0.039
≤60	14	30	9	
>60	40	34	9	
Stage				0.770
pT2c	0	1	0	
pT3b	2	1	1	
LpT1	14	22	9	
pT2a	6	6	1	
pT2b	2	4	0	
pT3a	3	2	2	
HpT1	12	8	2	
LpTa	9	14	2	
pT4a	1	4	1	
Surgical margin				0.647
Positive	2	3	0	
Negative	52	61	18	

The genotype rs4938723 polymorphism of pri-miR-34b/c in controls and cases were in HWE (χ^2^ = 2.483, *P* = 0.115 and χ^2^ = 0.019, *P* = 0.88, respectively).

## Discussion

In the present study, for the first time, we inspected whether the pri-miR-34b/c rs4938723 *t*>C polymorphism modifies the risk of bladder cancer in a sample from the Iranian population. The results showed that rs4938723 variant of pri-miR-34b/c was not associated with the risk of bladder cancer. As shown in ***Table 3***, several preceding studies have investigated the association between pri-miR-34b/c rs4938723 polymorphism and cancer risk in some populations and various types of cancer with inconsistent findings. It has been shown that rs4938723 variant was not associated with the risk of breast cancer (BC)^[37]^ and retinoblastoma^[38]^. The variant has been shown to be associated with increased risk of papillary thyroid carcinoma (PTC)^[27]^ and nasopharyngeal carcinoma^[39]^. The rs4938723 variant was found to be associated with increased risk of hepatocellular carcinoma (HCC) in the Chinese^[23,40–41]^ and Korean populations^[42]^.

**Tab.3:** Genotype distribution of miR-34b/c rs4938723 *t*>C among various studies and association with risk of cancer

Study	Country	Cancer type	Case/Control	Cases			Controls			Association
				TT	TC	CC	TT	TC	CC	
Chen *et al.*[^27^]	China	Papillary thyroid carcinoma	787/1,006	271	402	111	456	451	99	Increased risk
Li *et al.*[^39^]	China	Nasopharyngeal carcinoma	217/360	82	104	31	168	155	37	Increased risk
Liu *et al.*[^40^]	China	Hepatocellular carcinoma	164/305	63	80	21	152	141	13	Increased risk
Xu *et al.*[^23^]	China	Hepatocellular carcinoma	501/548	204	236	62	266	229	54	Increased risk
Chen *et al.*[^41^]	China	Hepatocellular carcinoma	286/572	102	146	38	272	267	33	Increased risk
Son *et al.*[^42^]	Korea	Hepatocellular carcinoma	157/201	69	75	13	110	74	17	Increased risk
Pan *et al.*[^28^]	China	Gastric cancer	197/289	102	76	19	121	137	31	Decreased risk
Yang *et al.*[^43^]	China	Gastric cancer	419/402	193	186	40	156	184	62	Decreased risk
Wu *et al.*[^44^]	China	Gastric cancer	897/992	405	396	92	476	430	84	No association
Zhang *et al.*[^45^]	China	Esophageal squamous cell carcinoma	1,109/1,275	489	536	84	569	573	133	Decreased risk
Zhu *et al.*[^24^]	China	Esophageal squamous cell carcinoma	237/274	113	99	25	122	122	30	No association
Oh *et al.*[^46^]	Korea	Colorectal cancer	545/428	272	233	40	216	171	41	No association
Gao *et al.*[^47^]	China	Colorectal cancer	347/488	175	144	28	216	210	62	Decreased risk
Yuan *et al.*[^32^]	China	Cervical cancer	328/568	117	157	36	242	258	68	Increased risk
Zhang *et al.*[^20^]	China	Renal cell carcinoma	710/760	302	324	84	352	344	64	Increased risk
Carvalho *et al.*[^38^]	Brazil	Retinoblastoma	130/105	52	64	14	45	44	16	No association
Sanaei *et al.*[^37^]	Iran	Breast cancer	263/221	125	115	23	100	106	15	No association
Hashemi *et al.*[^48^]	Iran	Prostate cancer	151/152	85	56	10	109	38	5	Increased risk
Tong *et al.*[^49^]	China	Childhood ALL	570/673	245	281	35	301	296	76	Decreases risk
Hashemi *et al.*[^50^]	Iran	Childhood ALL	110/120	77	31	2	62	52	6	Decreased risk
Current study	Iran	Bladder cancer	136/144	54	64	18	64	70	10	No association

Pan *et al.* and Yang *et al. * have found that pri-miR-34b/c rs4938723 variants significantly decreased the risk of gastric cancer (GC) in Chinese population^[28,43]^. On the other hand, the findings of Wu *et al. * did not support an association between the variant and risk of GC in Chinese population^[44]^. Zhang *et al.* findings revealed that rs4938723 variant significantly decreased the risk of esophageal squamous cell carcinoma (ESCC) in the Chinese population^[45]^. While, Zhu *et al.* has found no significant association between the variant and risk of ESCC in the Chinese population^[24]^.

Oh *et al.* have found no significant association between rs4938723 variant and colorectal cancer (CRC) in Korean population, while Gao *et al.* reported that this variant decrease the risk of CRC in Chinese population^[46–47]^. The rs4938723 variant have been shown to be associated with increased risk of cervical cancer^[32]^, renal cell carcinoma^[20]^ and prostate cancer^[48]^.

Tong *et al.*^[49]^ and Hashemi *et al.*^[50]^ reported that the rs4938723 variant significantly decreases the risk of childhood acute lymphoblastic leukemia (ALL).

There is no clear reason for the inconsistent findings regarding the association between pri-miR-34b/c rs4938723 variant and cancer risk. Ethnic, genetic, and/or environmental factors as well as gene-diet interaction may interact in various modes to either increase or decrease the risk of various cancers in different regions.

In summary, our findings did not support an association between rs4938723 polymorphism in the promoter region of pri-miR-34b/c and the risk of bladder cancer in a sample from the Iranian population. Further large-scale studies with diverse ethnicities are warranted to reveal the impact of rs4938723 on bladder cancer.

## References

[1] Siegel RL, Miller KD, Jemal A. Cancer statistics, 2016[J]. *CA Cancer J Clin*, 2016, 66(1): 7–30 . 26742998 10.3322/caac.21332

[2] Shiels MS, Gibson T, Sampson J, Cigarette smoking prior to first cancer and risk of second smoking-associated cancers among survivors of bladder, kidney, head and neck, and stage I lung cancers[J]. *J ClinOncol*, 2014, 32(35): 3989–3995 . 25385740 10.1200/JCO.2014.56.8220 PMC4251962

[3] Vermeulen SH, Hanum N, Grotenhuis AJ, Recurrent urinary tract infection and risk of bladder cancer in the Nijmegen bladder cancer study[J]. *Br J Cancer*, 2015, 112(3): 594–600 . 25429525 10.1038/bjc.2014.601 PMC4453642

[4] Burger M, Catto JW, Dalbagni G, Epidemiology and risk factors of urothelial bladder cancer[J]. *EurUrol*, 2013, 63(2): 234–241 . 22877502 10.1016/j.eururo.2012.07.033

[5] Giedl J, Rogler A, Wild A, TERT core promotor mutations in early-onset bladder cancer[J]. *J Cancer*, 2016, 7(8): 915–920 . 27313781 10.7150/jca.15006 PMC4910583

[6] Sankhwar M, Sankhwar SN, Bansal SK, Polymorphisms in the XPC gene affect urinary bladder cancer risk: a case-control study, meta-analyses and trial sequential analyses[J]. *Sci Rep*, 2016, 6: 27018 . 27246180 10.1038/srep27018 PMC4887911

[7] Hua Q, Lv X, Gu X, Genetic variants in lncRNA H19 are associated with the risk of bladder cancer in a Chinese population[J]. *Mutagenesis*, 2016, 31(5): 531–538 . 27091055 10.1093/mutage/gew018

[8] Aben KK, Witjes JA, Schoenberg MP, Familial aggregation of urothelial cell carcinoma[J]. *Int J Cancer*, 2002, 98(2): 274–278 . 11857419 10.1002/ijc.10191

[9] Bartel DP. MicroRNAs: genomics, biogenesis, mechanism, and function[J]. *Cell*, 2004, 116(2): 281–297 . 14744438 10.1016/s0092-8674(04)00045-5

[10] Esquela-Kerscher A, Slack FJ. Oncomirs—microRNAs with a role in cancer[J]. *Nat Rev Cancer*, 2006, 6(4): 259–269 . 16557279 10.1038/nrc1840

[11] He L, Thomson JM, Hemann MT, A microRNA polycistron as a potential human oncogene[J]. *Nature*, 2005, 435(7043): 828–833 . 15944707 10.1038/nature03552 PMC4599349

[12] Voorhoeve PM, le Sage C, Schrier M, A genetic screen implicates miRNA-372 and miRNA-373 as oncogenes in testicular germ cell tumors[J]. *Cell*, 2006, 124(6): 1169–1181 . 16564011 10.1016/j.cell.2006.02.037

[13] Li YQ, Lu JH, Bao XM, MiR-24 functions as a tumor suppressor in nasopharyngeal carcinoma through targeting FSCN1[J]. *J ExpClin Cancer Res*, 2015, 34(1): 130 . 26503504 10.1186/s13046-015-0242-6 PMC4621856

[14] Ruoming W, Zhen Y, Tengteng Z, Tumor suppressor microRNA-31 inhibits gastric carcinogenesis by targeting Smad4 and SGPP2[J]. *Cancer Gene Ther*, 2015, 22(12): 564–572 . 26494556 10.1038/cgt.2015.41

[15] Glover AR, Zhao JT, Gill AJ, MicroRNA-7 as a tumor suppressor and novel therapeutic for adrenocortical carcinoma[J]. *Oncotarget*, 2015, 6(34): 36675–36688 . 26452132 10.18632/oncotarget.5383 PMC4742203

[16] Ryan BM, Robles AI, Harris CC. Genetic variation in microRNA networks: the implications for cancer research[J]. *Nat Rev Cancer*, 2010, 10(6): 389–402 . 20495573 10.1038/nrc2867 PMC2950312

[17] Zhu S, Wu H, Wu F, MicroRNA-21 targets tumor suppressor genes in invasion and metastasis[J]. *Cell Res*, 2008, 18(3): 350–359 . 18270520 10.1038/cr.2008.24

[18] Buscaglia LE, Li Y. Apoptosis and the target genes of microRNA-21[J]. *Chin J Cancer*, 2011, 30(6): 371–380 . 21627859 10.5732/cjc.011.10132 PMC3319771

[19] Hashemi M, Sheybani-Nasab M, Naderi M, Association of functional polymorphism at the miR-502-binding site in the 3′ untranslated region of the SETD8 gene with risk of childhood acute lymphoblastic leukemia, a preliminary report[J]. *TumourBiol*, 2014, 35(10): 10375–10379 . 25048968 10.1007/s13277-014-2359-1

[20] Zhang S, Qian J, Cao Q, A potentially functional polymorphism in the promoter region of miR-34b/c is associated with renal cell cancer risk in a Chinese population[J]. *Mutagenesis*, 2014, 29(2): 149–154 . 24503183 10.1093/mutage/geu001

[21] Bossard P, Zaret KS. GATA transcription factors as potentiators of gut endoderm differentiation[J]. *Development*, 1998, 125(24): 4909–4917 . 9811575 10.1242/dev.125.24.4909

[22] Chou J, Provot S, Werb Z. GATA3 in development and cancer differentiation: cells GATA have it![J]. *J Cell Physiol*, 2010, 222(1): 42–49 . 19798694 10.1002/jcp.21943 PMC2915440

[23] Xu Y, Liu L, Liu J, A potentially functional polymorphism in the promoter region of miR-34b/c is associated with an increased risk for primary hepatocellular carcinoma[J]. *Int J Cancer*, 2011, 128(2): 412–417 . 20309940 10.1002/ijc.25342

[24] Zhu J, Yang L, You W, Genetic variation in miR-100 rs1834306 is associated with decreased risk for esophageal squamous cell carcinoma in Kazakh patients in northwest China[J]. *Int J Clin Exp Pathol*, 2015, 8(6): 7332–7340 . 26261633 PMC4525967

[25] Wang FJ, Ding Y, Mao YY, Associations between hsa-miR-603 polymorphism, lifestyle-related factors and colorectal cancer risk[J]. *Cancer Biomark*, 2014, 14(4): 225–231 . 24934365 10.3233/CBM-140395 PMC12928342

[26] Ji TX, Zhi C, Guo XG, MiR-34b/c rs4938723 polymorphism significantly decreases the risk of digestive tract cancer: meta-analysis[J]. *Asian Pac J Cancer Prev*, 2015, 16(14): 6099–6104 . 26320502 10.7314/apjcp.2015.16.14.6099

[27] Chen P, Sun R, Pu Y, Pri-Mir-34b/C and Tp-53 polymorphisms are associated with the susceptibility of papillary thyroid carcinoma: a case-control study[J]. *Medicine (Baltimore)*, 2015, 94(38): e1536 . 26402809 10.1097/MD.0000000000001536 PMC4635749

[28] Pan XM, Sun RF, Li ZH, Pri-miR-34b/c rs4938723 polymorphism is associated with a decreased risk of gastric cancer[J]. *Genet Test Mol Biomarkers*, 2015, 19(4): 198–202 . 25658980 10.1089/gtmb.2014.0287

[29] Nowakowska M, Płuciennik E, Wujcicka WI, The correlation analysis of WWOX expression and cancer related genes in neuroblastoma—a real time RT-PCR study[J]. *Acta Biochim Pol*, 2014, 61(1): 91–97 . 24455756

[30] Tao T, Chen S, Xu B, Association between hsa-miR-34b/c rs4938723 T>C promoter polymorphism and cancer risk: a meta-analysis based on 6,036 cases and 6,204 controls[J]. *Chin J Cancer Res*, 2014, 26(3): 315–322 . 25035659 10.3978/j.issn.1000-9604.2014.06.18 PMC4076713

[31] Liang TJ, Liu HJ, Zhao XQ, Lack of association of MiR-34b/c polymorphism (rs4938723) with hepatocellular carcinoma: a meta-analysis[J]. *PLoS One*, 2013, 8(7): e68588 . 23935875 10.1371/journal.pone.0068588 PMC3729562

[32] Yuan F, Sun R, Chen P, Combined analysis of pri-miR-34b/c rs4938723 and TP53 Arg72Pro with cervical cancer risk[J]. *Tumour Biol*, 2016, 37(5): 6267–6273 . 26619844 10.1007/s13277-015-4467-y

[33] Tong N, Chu H, Wang M, Pri-miR-34b/c rs4938723 polymorphism contributes to acute lymphoblastic leukemia susceptibility in Chinese children[J]. *Leuk Lymphoma*, 2016, 57(6): 1436–1441 . 26380954 10.3109/10428194.2015.1092528

[34] Bensen JT, Tse CK, Nyante SJ, Association of germline microRNA SNPs in pre-miRNA flanking region and breast cancer risk and survival: the Carolina Breast Cancer Study[J]. *Cancer Causes Control*, 2013, 24(6): 1099–1109 . 23526039 10.1007/s10552-013-0187-z PMC3804004

[35] Hashemi M, Bizhani F, Danesh H, MiR-608 rs4919510 C>G polymorphism increased the risk of bladder cancer in an Iranian population[J]. *Aims Genet*, 2016, 3(4): 212–218.

[36] Hashemi M, Hanafi Bojd H, EskandariNasab E, Association of adiponectin rs1501299 and rs266729 gene polymorphisms with nonalcoholic fatty liver disease[J]. *Hepat Mon*, 2013, 13(5): e9527 . 23922565 10.5812/hepatmon.9527 PMC3734897

[37] Sanaei S, Hashemi M, Rezaei M, Evaluation of the pri-miR-34b/c rs4938723 polymorphism and its association with breast cancer risk[J]. *Biomed Rep*, 2016, 5(1): 125–129 . 27347415 10.3892/br.2016.690 PMC4906800

[38] Carvalho IN, Reis AH, Dos Santos AC, A polymorphism in mir-34b/c as a potential biomarker for early onset of hereditary retinoblastoma[J]. *Cancer Biomark*, 2017, 18(3): 313–317 . 28106538 10.3233/CBM-160248 PMC13020604

[39] Li L, Wu J, Sima X, Interactions of miR-34b/c and TP-53 polymorphisms on the risk of nasopharyngeal carcinoma[J]. *Tumour Biol*, 2013, 34(3): 1919–1923 . 23504554 10.1007/s13277-013-0736-9

[40] Liu CJ, Ma XW, Zhang XJ, pri-miR-34b/c rs4938723 polymorphism is associated with hepatocellular carcinoma risk: a case-control study in a Chinese population[J]. *Int J Mol Epidemiol Genet*, 2017, 8(1): 1–7 . 28337312 PMC5344989

[41] Chen LL, Shen Y, Zhang JB, Association between polymorphisms in the promoter region of pri-miR-34b/c and risk of hepatocellular carcinoma[J]. *Genet Mol Res*, 2016, 15(4) . 27808368 10.4238/gmr.15048723

[42] Son MS, Jang MJ, Jeon YJ, Promoter polymorphisms of pri-miR-34b/c are associated with hepatocellular carcinoma[J]. *Gene*, 2013, 524(2): 156–160 . 23632240 10.1016/j.gene.2013.04.042

[43] Yang C, Ma X, Liu D, Promoter polymorphisms of miR-34b/c are associated with risk of gastric cancer in a Chinese population[J]. *Tumour Biol*, 2014, 35(12): 12545–12554 . 25190020 10.1007/s13277-014-2574-9

[44] Wu Y, Jia Z, Cao D, Predictive value of MiR-219–1, MiR-938, MiR-34b/c, and MiR-218 polymorphisms for gastric cancer susceptibility and prognosis[J]. *Dis Markers*, 2017, 2017 4731891. 10.1155/2017/4731891 PMC5337385 28298809

[45] Zhang J, Huang X, Xiao J, Pri-miR-124 rs531564 and pri-miR-34b/c rs4938723 polymorphisms are associated with decreased risk of esophageal squamous cell carcinoma in Chinese populations[J]. *PLoS One*, 2014, 9(6): e100055 . 24945256 10.1371/journal.pone.0100055 PMC4063769

[46] Oh J, Kim JW, Lee BE, Polymorphisms of the pri-miR-34b/c promoter and TP53 codon 72 are associated with risk of colorectal cancer[J]. *Oncol Rep*, 2014, 31(2): 995–1002 . 24337371 10.3892/or.2013.2926

[47] Gao LB, Li LJ, Pan XM, A genetic variant in the promoter region of miR-34b/c is associated with a reduced risk of colorectal cancer[J]. *Biol Chem*, 2013, 394(3): 415–420 . 23183747 10.1515/hsz-2012-0297

[48] Hashemi M, Danesh H, Bizhani F, Pri-miR-34b/c rs4938723 polymorphism increased the risk of prostate cancer[J]. *Cancer Biomark*, 2017, 18(2): 155–159 . 27983526 10.3233/CBM-160058 PMC13020591

[49] Tong N, Chu H, Wang M, Pri-miR-34b/c rs4938723 polymorphism contributes to acute lymphoblastic leukemia susceptibility in Chinese children[J]. *Leuk Lymphoma*, 2015, 57(6): 1436–1441 . 26380954 10.3109/10428194.2015.1092528

[50] Hashemi M, Bahari G, Naderi M, Pri-miR-34b/c rs4938723 polymorphism is associated with the risk of childhood acute lymphoblastic leukemia[J]. *Cancer Genet*, 2016, 209(11): 493–496 . 27886674 10.1016/j.cancergen.2016.09.009

